# Dipole Potential of Monolayers with Biologically Relevant Lipid Compositions

**DOI:** 10.3390/molecules29245843

**Published:** 2024-12-11

**Authors:** Renato M. S. Cardoso, Fabiana Lairion, Edgardo Anibal Disalvo, Luís M. S. Loura, Maria João Moreno

**Affiliations:** 1 Coimbra Chemistry Center, Institute of Molecular Sciences (CQC-IMS), University of Coimbra, 3004-535 Coimbra, Portugal; 2 Chemistry Department, Faculty of Science and Technology, University of Coimbra, 3004-535 Coimbra, Portugal; 3 Institute of Biochemistry and Molecular Medicine Prof. Alberto Boveris (IBIMOL), University of Buenos Aires and National Council for Scientific and Technical Research (CONICET), Buenos Aires 1113, Argentina; 4 Applied Biophysics and Food Research Center (CIBAAL), National University of Santiago del Estero and National Council for Scientific and Technical Research (CONICET), Santiago del Estero 4206, Argentina; 5 Faculty of Farmacy, University of Coimbra, 3000-548 Coimbra, Portugal

**Keywords:** transmembrane potential, cholesterol, POPC, POPE, POPS, surface pressure, monolayers, area per lipid

## Abstract

The membrane dipole potential that arises from the interfacial water and constitutive dipolar groups of lipid molecules modulates the interaction of amphiphiles and proteins with membranes. Consequently, its determination for lipid mixtures resembling the existing diversity in biological membranes is very relevant. In this work, the dipole potentials of monolayers, formed at the air–water interface, from pure or mixed lipids (1-palmitoyl-2-oleoyl-*sn*-glycero-3-phosphocholine (POPC), 1-palmitoyl-2-oleoyl-*sn*-glycero-3-phosphoethanolamine (POPE), 1-palmitoyl-2-oleoyl-*sn*-glycero-3-phosphatidyserine (POPS), sphingomyelin (SpM) and cholesterol) were measured and correlated with the mean area per lipid. The results showed that, as previously observed, cholesterol increases the dipole potential in correspondence with the decrease in the average area per lipid. At the small mole fractions encountered in biomembranes, the presence of the negatively charged lipid POPS increases the dipole potentials of monolayers despite inducing an increase in the average area per lipid. Additionally, the inclusion of POPE in POPC:cholesterol monolayers disrupts the area condensation induced by cholesterol while increasing the membrane dipole moment, leading to a small reduction in the dipole potential. This trend is reinforced for the quaternary POPC:cholesterol:POPE:POPS 4:3:2:1 system, which mimics the inner leaflets of eukaryotic plasma membranes. In agreement with previous works, the replacement of phosphocholine lipids with sphingomyelin leads to a decrease in the dipole potential. Together, this results in a lower dipole potential for the SpM-enriched outer leaflet, generating a non-zero transbilayer dipole potential in the asymmetric plasma membranes of eukaryotic cells.

## 1. Introduction

The total electric profile of a membrane may be defined by three distinct types of electrostatic potential: the transmembrane potential, the surface charge potential and the dipole potential [1,2,3,4]. The first is a consequence of the charge displacement from one side to the other of a membrane, due to a difference in the ion concentrations between both aqueous compartments. The surface charge potential, which is described by the Gouy–Chapman theory, results from the presence of charged groups in the lipids and the ion distribution in the electrical double layer on the membrane surface. The third, which is no less important, originates from the preferred alignment of water molecules and certain constitutive dipoles in lipids. The dipole potential was first described by Liberman and Topaly in 1969, through membrane conductivity changes upon the addition of large hydrophobic anions and cations (TPB^−^, TPP^+^). They observed that the membrane conductance was much larger for hydrophobic anions than for cations. This difference was attributed to their distinct partitioning into the center of the membrane, and they assumed that the presence of a more positive potential within the membrane was the main reason for the permeability differences [5]. The main sources contributing to a membrane’s internal dipole potential are (i) the lipid carbonyl groups, (ii) the dipole resulting from the phospholipid headgroup (choline–phosphate dipole in the case of phosphatidylcholines), (iii) the terminal methyl group in the lipid alkyl chain and (iv) interfacial water hydrating the phospholipid headgroups [1,2,3,4,6,7,8,9,10]. Studies in monolayers with phosphatidylcholines revealed a dipole potential of approximately 400 mV. This potential generates a considerably larger membrane electric field than that of the surface charge or the transmembrane potentials, suggesting that it has an important biological role. In fact, the binding affinity or the orientation of a membrane peptide was shown to be affected by the dipole potential [11,12]. We have recently demonstrated that the dipole potential exerts an important influence on the association of amphiphiles with ordered lipid membranes, changing their membrane affinity and transversal location, with effects on amphiphile aggregation and ionization [13]. The effect of the dipole potential on the permeability of charged molecules is the basis of its identification, and this has been observed by several authors [14,15]. Molecular dynamics simulations have shown that this effect is relevant even for uncharged polar molecules, with the dipole moment of the permeating molecule re-orienting as it moves across the bilayer [16]. The dipole potential has also been recognized as a modulator of the activity of membrane-embedded peptides [17] and of the kinetics of charge transfer reactions in reaction centers [18]. It was shown in Monte Carlo simulations that the dipole potential is also an important parameter in phase miscibility and topology [19].

The dipole potentials of lipid bilayers, between the membrane–water interface and the bilayer center, cannot be measured directly, and this has hindered their characterization. Their effect on the relative permeability of hydrophobic cations and anions is primarily considered, but this method is tedious and not free from artifacts. More recently, several fluorescent probes that are sensitive to the local electrical field have been developed [20], and cryoelectron microscopy (cryoEM) has been used to characterize the dipole potential profile in rapidly frozen liposomes [21]. However, the use of fluorescent probes to quantitatively characterize the dipole potential relies on calibration with other methodologies, and the interpretation of cryoEM rests on several assumptions (further details may be found in references [22,23]).

The simplest and most commonly used methodology to quantitatively characterize the dipole potential is the use of lipid monolayers. Briefly, this consists of measuring the potential difference across an air–water interface, first in the absence and then in the presence of a formed lipid monolayer [6,24].

The results of monolayer studies and of the relative permeability of hydrophobic ions have shown that the dipole potential can be changed through the addition of certain dipolar molecules, such as phloretin [25], cholesterol and its analog, 6-ketocholestanol [14,26,27,28]. Sterols are present in large quantities in the biological membranes of eukaryotic cells, and the effect of cholesterol on the membrane dipole moment was first addressed by Szabo in 1974 [14]. Using hydrophobic ions as molecular probes, it was observed that, upon the addition of cholesterol to a neutral membrane, there was a 30-fold increase in anion permeability, compared to a 100-fold decrease in cation permeability. This distinct behavior for anions and cations was compatible with an increase in the dipole potentials of cholesterol-containing membranes. Later, this observation was confirmed through measurements of the dipole potentials of cholesterol-containing egg phosphatidylcholine (EggPC) monolayers [28]. The main reason why cholesterol increases the membrane potential is by enhanced lipid packing in the membrane, the so-called cholesterol condensation effect [29], although changes in the alignment of interfacial water are also involved [30,31].

Biological membranes are composed of several types of lipids, whose relative proportions vary significantly among distinct cells and within cell membranes. Additionally, the distribution of the lipids in both bilayer leaflets is not homogeneous, depending on the chemical environment that is in direct contact with each monolayer [32] and on the asymmetric synthesis and active translocation by membrane lipids and proteins [33]. Typically, the erythrocyte membrane contains 30–50% cholesterol (Chol), 15–20% sphingomyelin (SpM) and 40–50% glycerophospholipids, being 20–25% phosphatidylcholines (PC), 10–25% phosphatidylethanolamines (PE) and 5–15% of phosphatidylserine (PS) [33,34,35,36,37]. While SpM, PC and Chol are the major components of the outer exoplasmic leaflets of the plasma membranes in eukaryotic cells, the composition of the inner leaflet is mainly PC, PE and PS [33,34,38].

The dipole potentials of phosphatidylcholines, sphingomyelin and their mixtures with cholesterol have been widely discussed in the literature, seeking to obtain insights into the sources contributing to the dipole potential [39,40,41,42,43,44]. The quantification of the membrane dipole potential with different lipid compositions is also important in order to understand its role in the partitioning and permeation of amphiphilic molecules, especially those having a preferential dipole moment orientation. In the last few years, our group has characterized the interactions of different amphiphiles and drugs with membranes of varying lipid composition [45,46,47,48,49,50], and the magnitude of the dipole potential has emerged as a membrane property that may play a crucial role [13,51]. Moreover, changes in the membrane dipole potential have been proposed to explain some unspecific drug effects [52,53,54,55,56,57,58]. In this respect, the different dipole potentials of distinct biomembranes influence the drug–membrane affinity and localization, and the presence of the drug affects the membrane’s dipole potential, with an impact on the structures and functions of membrane-associated proteins [59,60].

In spite of its importance, the dipole potential in biologically relevant lipid mixtures has not been systematically addressed in the literature. In this work, we fill this gap via the characterization of the effects of the headgroups PC, PE and PS, at biologically relevant molar fractions, as well as their mixtures with cholesterol. The systems used are monolayers formed at the air–water interface, allowing the direct characterization of the dipole potential.

## 2. Results

### 2.1. Area per Lipid in Monolayers

**Figure 1:**
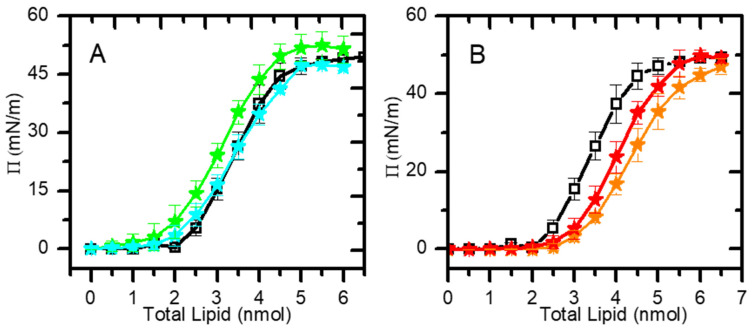
Formation of lipid monolayers at 25 ± 1 °C, followed by changes in surface pressure as a function of the amount of lipid added. (**A**) Pure POPC (□); POPC:POPS (9:1, molar ratio) (★); POPC:POPE (8:2) (★). (**B**) Pure POPC (□); POPC:CHOL:POPE (5:3:2) (★); POPC:CHOL:POPE:POPS (4:3:2:1) (★). The lines are a guide to the eye.

The area per lipid allows us to quantitatively evaluate differences in lipid packing within a monolayer and to correlate them with the membrane dipole potential produced by mixing different lipids. Moreover, together with the results of the dipole potential, this allows the rationalization of the effects (condensation/expansion/reorientation) of different lipids in more complex lipid mixtures. In this work, we have calculated the area per lipid from the changes in the surface pressure as a function of the amount of lipid added to the air–water interface (Figure 1). The chosen lipid compositions mimic the lipid bilayers of distinct biological membranes of eukaryotic cells (for which data are not available in the literature), as well as simpler mixtures required for the interpretation of the final results. As a reference, we have also measured the area per lipid in the well-characterized monolayer of pure POPC.

**Figure 2:**
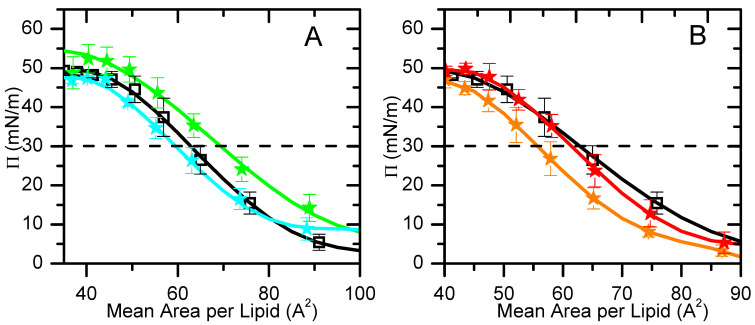
Mean area per lipid isotherm for POPC (□), POPC:POPS (90:10) (★) and POPC:POPE (80:20) (★) (**A**) and for POPC:CHOL:POPE (5:3:2) (★) and POPC:CHOL:POPE:POPS (4:3:2:1) (★) (**B**). The solid curves are the best polynomial fits for areas smaller than 100 Å^2^ in the liquid expanded state. The dashed horizontal line indicates *π* = 30 mN/m.

The mean area per lipid values most commonly reported in the literature are those at 30 mN/m, which is assumed to correspond to the surface pressure of a lipid bilayer [61]. In order to analyze our results compared with previously reported data, a polynomial equation was fitted to the experimental π-area isotherms, for areas smaller than 100 Å^2^ (liquid expanded/condensed state) (Figure 2), and the area per lipid at a surface pressure of 30 mN/m was calculated; see Table 1. The π-area isotherm profile and the area per lipid obtained for pure POPC at *π*_sat_ and 30 mN/m are similar to the values obtained experimentally [24,62,63] and by molecular dynamics simulations [64]. The addition of 20 mol% POPE changes the *π*-area isotherm to smaller areas, decreasing the area per lipid, both at 30 mN/m and at the saturation pressure, in agreement with the smaller area observed for pure POPE monolayers [24]. In the presence of 10% of POPS, the *π*-area isotherm is shifted both to larger areas and pressures, leading to a significantly larger area per lipid at 30 mN/m. It should, however, be noted that, at the saturation pressure, the area per lipid is similar to that of pure POPC, indicating that the electrostatic repulsion between the negatively charged serine groups is somewhat compensated for by lipid–lipid interactions at pressures near saturation. The replacement of 30 mol% POPC with cholesterol in the POPC:POPE (8:2) monolayer leads to a significant decrease in the area at 30 mN/m but little effect at saturation. This is in contrast with the effect observed in pure POPC, where the monolayer is condensed at all pressures due to the presence of cholesterol. Finally, the quaternary mixture, which mimics the inner leaflet of a plasma membrane, showed an area per lipid equal to that of pure POPC monolayers at saturation and slightly smaller at 30 mN/m. The comparison of the *π*-area isotherm profiles of POPC:CHOL:POPE and POPC:CHOL:POPE:POPS shows that the inclusion of the charged lipid shifts the curve to larger areas and pressures. This suggests that the condensation effect induced by cholesterol and POPE is compensated for by the electrostatic repulsion between the charged POPS.

### 2.2. Dipole Potential in Lipid Monolayers

**Figure 3:**
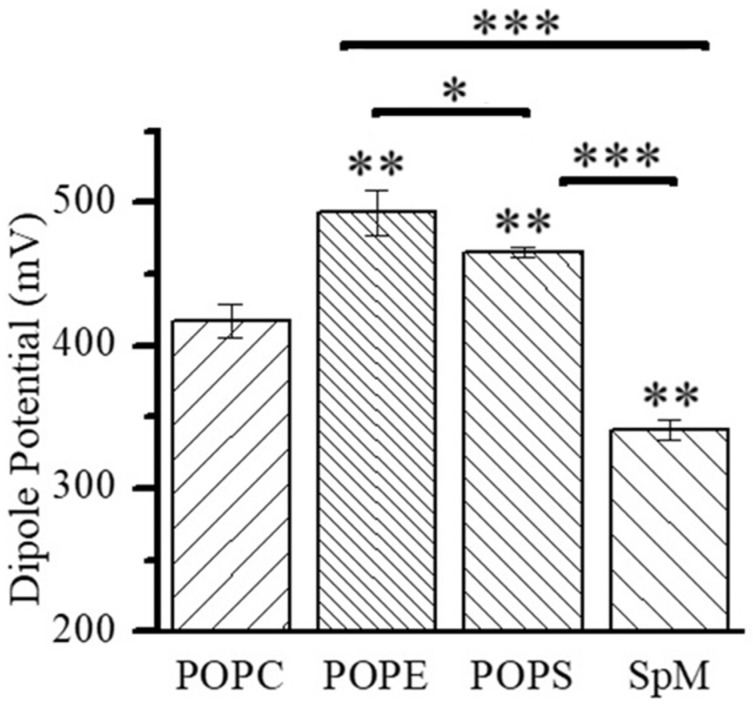
Dipole potential in pure POPC, POPE, POPS and SpM monolayers at *π*_sat_ with the aqueous buffer as a subphase. The asterisk represents the statistical significance of the differences between POPE and POPS, *p* < 0.05. The double asterisk represents the statistical significance of the differences from POPC, *p* < 0.001. The triple asterisk represents the statistical significance of the differences from SpM, *p* < 10^−6^.

The magnitude of the dipole potential depends on the orientation and density of the dipoles present in the lipid monolayers, and this parameter is important in understanding the lipid organization in bilayers [68], as well as the interactions established with and the functions of membrane-associated molecules [13,54,58]. The comprehension of the dipole potential variation in biologically relevant lipid mixtures requires a primary analysis of less complex mixtures and pure lipids. Consequently, the dipole potentials of pure POPC, POPE, SpM and POPS in monolayers formed at the air–water interface were obtained first (Figure 3). In agreement with other results for negatively charged lipids [67,69], the measured potential for POPS is lower than the dipole potential due to the surface potential (Equation (3)), and, for these monolayers, we estimated the surface dipole potential using the Gouy–Chapman theory (Equation (4)). The contribution of the surface potential to the measured potential in pure POPS monolayers is 135 mV, considering an area similar to POPC (49.4 Å^2^) and a formal charge of −1 per lipid, which generates a surface charge density (σ) of −3.1 C/m^2^ [70].

The results show, in agreement with other monolayer studies [24] and molecular dynamics simulations [71], that ethanolamine and serine headgroups promote an increase in the dipole potential when compared to choline. As observed previously, the dipole potential of SpM monolayers is significantly smaller than that of POPC despite the same phosphate–choline headgroup [72].

The increase in dipole potential in the POPE and POPS monolayers is in parallel with an increase in lipid packing. In contrast, a much smaller dipole potential is obtained for SpM despite higher lipid packing, as observed by other authors [73,74]. The SpM alkyl chain’s motional constraints and its ability to establish a network of hydrogen bonds between headgroups increases the lipid packing in these membranes [75,76,77]. However, this increase in packing does not lead to an increase in the dipole potential. Thus, the dipole potential is influenced by structural differences due to the presence of an OH in SpM, instead of the carbonyl groups in POPC, and/or due to the interfacial hydration water layer.

**Figure 4:**
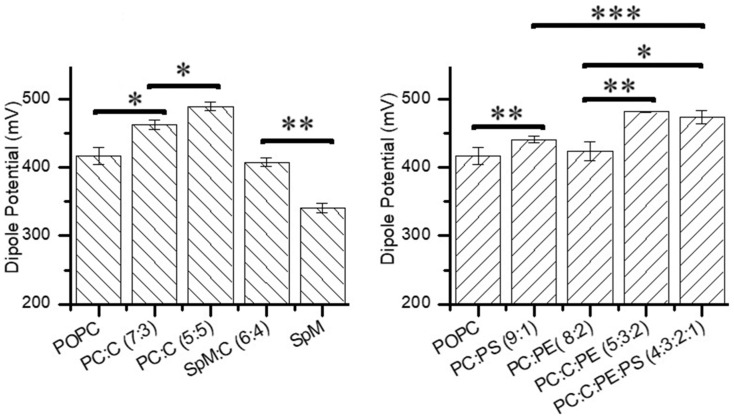
Dipole potential in monolayers composed of lipid mixtures, where PC, PS, PE, C and SpM abbreviations refer to POPC, POPS, POPE, Chol and Egg-SpM, respectively. The lipid molar proportion in the mixtures is given in parentheses. In the left plot, the asterisk represents the statistical significance of differences between POPC and PC:C 7:3, as well as between the latter and PC:C 5:5, *p* < 0.001, while the double asterisk represents the statistical significance of the difference between SpM and SpM:C 6:4, *p* < 10^−4^. In the right plot, the asterisk represents the statistical significance of the difference between PC:PE 8:2 and PC:C:PE:PS 4:3:2:1, *p* < 0.05, while the double asterisk represents the statistical significance of the differences between POPC and PC:PS 9:1, as well as between PC:PE 8:2 and PC:C:PE 5:3:2, *p* < 0.01, and the triple asterisk represents the statistical significance of the difference between PC:PS 9:1 and PC:C:PE:PS 4:3:2:1, *p* < 10^−5^.

The addition of increasing percentages of cholesterol to POPC increases the dipole potential from 417 mV to ~490 mV, at 50% cholesterol, which has been attributed to the increase in membrane lipid packing in these membranes [66,73] (Figure 4). This dipole potential variation (17%) is in agreement with the observed difference for EggPC:cholesterol monolayers at equimolar concentrations [28]. The addition of 40 mol% cholesterol to SpM leads to a 20% increase in the dipole potential, in agreement with the 26% increase observed in brain SpM at 50 mol% cholesterol [72]. However, the larger increase observed in the dipolar potential of SpM:Chol monolayers is not sufficient to approach the dipole potential of POPC:CHOL monolayers (at 30 or 50 mol% cholesterol). It is noteworthy that, despite the distinct physical state of pure POPC and SpM monolayers (liquid disordered and gel, respectively), and the corresponding much larger area per lipid of the former, the area per lipid in POPC and SpM monolayers saturated with cholesterol is similar. The dipole potential of the cholesterol-saturated monolayers is, however, much smaller in SpM:Chol.

The addition of biologically relevant amounts of POPS (10%) and POPE (20%) to POPC leads to a small increase in the dipole potential. Surprisingly, despite the much larger dipole potential of pure POPE and the opposite variations in the mean area per lipid, the increase due to the addition of 20 mol% POPE is smaller than that generated by the addition of 10 mol% POPS. This can only be understood in terms of the larger dipole moment of POPS when present at small molar fractions in POPC mixed monolayers as compared with pure POPS monolayers, and it is in agreement with an increase in the ionization constant of POPS at high surface charge densities [78,79].

Upon the addition of POPE (20%), and in the presence of cholesterol, there is a dipole potential increase of approximately 4%, from 463 mV in POPC:CHOL (7:3) to 482 mV in POPC:CHOL:POPE (5:3:2). The replacement of 10 mol% POPC with POPS in this ternary mixture, representing the inner leaflet of a plasma membrane, leads to a small decrease in the dipole potential, from 482 to 474 mV. This is surprising in regard to the increase in the dipole potential observed at 10 mol% POPS in POPC and raises questions regarding the lateral homogeneity of this lipid composition.

The dipole potentials shown in Table 1 and discussed above correspond to monolayers at the saturation pressure. Although this is the condition at which the lipids in the monolayer are in equilibrium with the bilayers in the aqueous phase, it is generally assumed that the properties of the bilayers are better described by monolayers at smaller lateral pressures. This is supported by comparisons of the mean area per lipid, which are smaller in monolayers at the saturation pressure than in bilayers, and by studies of partitioning into monolayers and bilayers, which show that partitioning into densely packed monolayers is hindered [80]. Comparisons of the main phase transition temperature in bilayers and monolayers at different lateral pressures have shown, using the method of monolayer compression, that the lateral pressure of monolayers at which they behave as bilayers is dependent on the lipid composition but is usually smaller than the saturation pressure [81]. It has, however, been shown that good correspondence between the temperature of the main phase transition in bilayers and in monolayers at the saturation pressure is obtained when the constant area method is followed [68], as in this work. Some controversy notwithstanding, it is usually considered that monolayers at a lateral pressure of 30 mN/m [61,82] are good models for bilayers [81].

Given that, at the saturation pressure and at 30 mN/m, the lipid monolayer is in the same phase, the orientation of the lipids should be approximately maintained, and only the density of the dipoles contributes to the observed changes in the dipole potential. According to this assumption, the dipole potential at 30 mN/m ($\psi_{d\left(30\;\mathrm{mN}/m\right)}^{*}$) was calculated using the Helmholtz equation for a parallel-plate capacitor [24]; see Equation (1).

(1) $$\Delta V=12\pi \mu_{⊥}/A$$

where ∆*V* is the dipole potential variation through the hydrated lipid monolayer (mV), *A* is the mean area per lipid (Å^2^) and *μ*_⊥_ is the component of the molecular dipole moment perpendicular to the lipid–water interface (mD).

Additionally, following the work of Smaby and Brockman [67], a modified equation was also considered; see Equation (2). These researchers observed the linear dependence of ΔV on 1/*A* for a number of lipid monolayers, albeit with a finite intercept $\Delta V_{0}$, which is considered in Equation (2).

(2) $$\Delta V=12\pi \mu_{⊥}/A+\Delta V_{0}$$

The experimental intercept values reported in reference [67] only concern pure lipid monolayers, of which POPC and POPE are also addressed here. Therefore, for most systems in the current study, no $\Delta V_{0}$ values are available for use in Equation (2). However, while there is dispersion in the recovered intercept values of reference [67], they hover around 100 mV for zwitterionic phospholipids. To evaluate the impact of this area-independent contribution to ΔV for the value of the dipole potential calculated at 30 mN/m, we have also calculated this property assuming $\Delta V_{0}$ = 100 mV for all lipid compositions.

**Figure 5:**
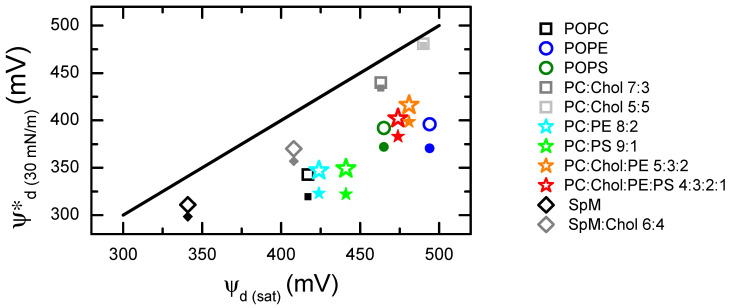
Dipole potentials of monolayers at a lateral pressure of 30 mN/m ($\psi_{d\left(30\;\mathrm{mN}/m\right)}^{*}$), calculated from Equation (1) (closed symbols) or Equation (2) with $\Delta V_{0}$ = 100 mV (open symbols), versus their dipole potentials measured at the saturation pressure ($\psi_{d\left(\mathrm{sat}\right)}$). The straight line represents the identity line ($\psi_{d\left(\mathrm{sat}\right)}$ = $\psi_{d\left(30\;\mathrm{mN}/m\right)}^{*}$). The lipid monolayers with $\psi_{d\left(30\;\mathrm{mN}/m\right)}^{*}$ closer to the identity line are the POPC:CHOL mixtures. Pure SpM lies also close to the identity line, while monolayers composed of pure POPC, POPE and POPS and their mixtures (including those with cholesterol) show the largest deviations. Consideration of Equation (2) with a typical area-independent contribution to the dipole potential, $\Delta V_{0}$ = 100 mV, leads to a slight increase in the calculated $\psi_{d\left(30\;\mathrm{mN}/m\right)}^{*}$, approaching $\psi_{d\left(\mathrm{sat}\right)}$.

The dipole potential at a lateral pressure of 30 mN/m, calculated from Equations (1) and (2) (the latter with $\Delta V_{0}$ = 100 mV), namely $\psi_{d\left(30\;\mathrm{mN}/m\right)}^{*}$, is represented in Figure 5 as a function of the surface potential measured at the saturation lateral pressure, $\psi_{d\left(\mathrm{sat}\right)}$. The $\psi_{d\left(30\;\mathrm{mN}/m\right)}^{*}$ values are also presented in Table 2, together with the corresponding transverse dipole moment components $\mu_{⊥}$.

Using Equation (1), a positive relation between the dipole potential at the two lateral pressures is observed, but the correlation is poor (*R*^2^ = 0.54 and 0.65 for $\Delta V_{0}$ = 0 or 100 mV, respectively). This results from the distinct area variations (at $\pi_{\mathrm{sat}}\;\mathrm{vs}.\;$30 mN/m) for the different lipid compositions. For the monolayers that present a small area variation at lateral pressures above 30 mN/m, the calculated $\psi_{d\left(30\;\mathrm{mN}/m\right)}^{*}$ is similar to the measured $\psi_{d\left(\mathrm{sat}\right)}$. This is the case for monolayers with a large compressibility modulus, such as SpM- and cholesterol-enriched membranes [66]. Lipid monolayers in a more expanded state are compressed more easily and show a large area variation at 30 mN/m and above. The value of $\psi_{d\left(30\;\mathrm{mN}/m\right)}^{*}$ is therefore much smaller than $\psi_{d\left(\mathrm{sat}\right)}$. Due to the smaller dependence of $\psi_{d}$ on the lipid area when considering the significant contribution of an area-independent potential (Equation (2)), the values of $\psi_{d\left(30\;\mathrm{mN}/m\right)}^{*}$ calculated with $\Delta V_{0}$ = 100 mV are closer to $\psi_{d\left(\mathrm{sat}\right)}$, especially for the more expanded systems with a significant variation in the area per lipid. This justifies the somewhat better correlation between the two dipole potentials (*R*^2^ = 0.65). Still, there is no significant overall qualitative difference between the two approaches. It is also pertinent to compare the values obtained here and shown in Table 2 for $\mu_{⊥}$ with those reported by Smaby and Brockman for POPC (468 mD), POPE (467 mD) and POPS (459 mD) [67]. While these values are of similar magnitude to those of the present work, they lie between the estimates obtained here using Equations (1) and (2) for each system. The relative differences between the values in Table 2 for these lipids and the corresponding ones in reference [67] lie within [16%, 19%] and [−12%, −6%] for estimation with Equation (1) or (2), respectively. The overall better agreement in the latter case is expected, because the previous authors also used Equation (2) to analyze their data, albeit with a $\Delta V_{0}$ value optimized for each system. In any case, these differences are reasonably small, given the distinct subphase compositions in the two studies and the fact that $\mu_{⊥}$ is not directly obtained from experiments but rather retrieved from the analysis of the dipole potential and area per lipid data.

On another note, the values calculated above for the dipole potential at 30 mN/m assume the same lipid orientation for monolayers at this lateral pressure and at saturation. While this approximation holds reasonably well for the more condensed systems (i.e., SpM and systems containing ≥30 mol% cholesterol), deviations from this behavior could be expected for more expanded monolayers. If the lipid reorganization accompanying the increased expansion at 30 mN/m were accompanied by a decrease in the transverse dipole moment $\mu_{⊥}$, the resulting $\psi_{d\left(30\;\mathrm{mN}/m\right)}^{*}$ would be lower, deviating further from $\psi_{d\left(\mathrm{sat}\right)}$. The fact that excellent linear dependences between ∆*V* and 1/*A* for lipid monolayers in the liquid expanded state were observed by Smaby and Brockman [67] for a large variety of lipids, including POPC and POPE, suggests that this effect might be relatively small.

## 3. Discussion

Our discussion is mainly centered around two observables: the average area per lipid *A* and the dipole moment $\mu_{⊥}$. Together, they determine the dipole potential, as described in Equations (1) and (2).

**Figure 6:**
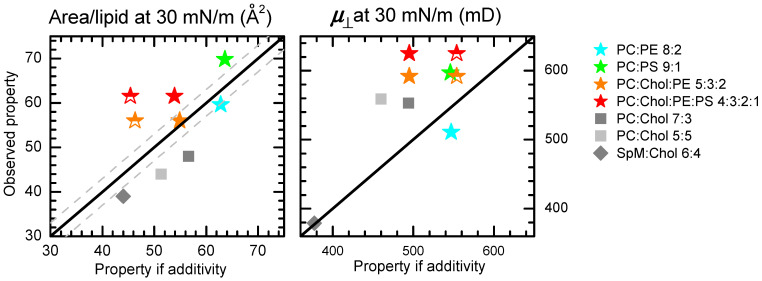
Average area per lipid *A* (**left**) and membrane dipole potential $\mu_{⊥}$ (**right**) for monolayers at a lateral pressure of 30 mN/m for mixed systems, represented as a function of the value calculated assuming the linear combination of the pure monolayer values (filled symbols). The linear replacement of the PC contribution in PC:Chol 7:3 by PE and/or PS (ternary and quaternary mixtures are also shown as half-filled stars with the corresponding color; see text for details). The straight line represents the identity line for the predicted property in both panels.

The area per lipid for pure POPC at 30 mN/m generally agrees with the experimental data available for the corresponding bilayer system, collected by Marsh [83], being significantly higher than those for PS and PE lipids with the same acyl chain composition. Regarding the mixed systems, the comparison between the area per lipid observed and that predicted on the basis of additive behavior is presented in Figure 6, left panel, for the distinct lipid compositions. The first observation is that the area for the POPC:CHOL and SpM:Chol mixtures (grey symbols) is always lower than that predicted (considering an area of 30 Å^2^ for cholesterol [73,84]), in agreement with the well-established condensing effect of cholesterol on phosphatidylcholine-containing lipids [29,73]. The area obtained for the POPC:POPE 8:2 monolayers (orange star) is not statistically different from that predicted when assuming additive behavior. In contrast, POPC:POPS monolayers (green star) are expanded when compared with the constituent pure lipids, possibly reflecting non-ideal mixing, as previously observed [85].

Two approaches were followed for the calculation of the predicted area in the case of the more complex ternary and quaternary mixtures. In the first approach, additivity in the area of all lipid components was considered, leading to the filled orange and red stars for PC:Chol:PE 5:3:2 and PC:Chol:PE:PS 4:3:2:1, respectively. The area predicted is in excellent agreement with that observed for the ternary mixture POPC:CHOL:POPE, strongly suggesting that cholesterol’s condensing effect is negligible in the presence of POPE. This behavior has been previously observed for DMPE:Chol binary mixtures at lateral pressures above 20 mN/m [86], with our results suggesting that the presence of significant amounts of phosphatidylethanolamine lipids are also able to suppress the condensing effect of cholesterol on POPC. When considering the quaternary mixture containing also POPS, the area observed is larger than that predicted. The deviation is similar to that observed in POPC:POPS, suggesting that it is being influenced by the same factors. In the second approach followed for the ternary and quaternary mixtures containing 30 mol% cholesterol, it was assumed that the POPC:CHOL area was the same as in the 7:3 binary mixture, and the area of substituted POPC was replaced by that of POPS and/or POPE. The results obtained are shown as half-filled stars. The areas per lipid predicted are notably lower than those measured, again suggesting that POPE is able to abolish the condensing effect of cholesterol on POPC.

The effects of POPE on the area per lipid observed in the complex mixtures may be understood as arising from a non-favorable interaction between POPE and cholesterol [86] (implying a lack of condensation of POPE acyl chains by the latter) and the competition of POPE with cholesterol for POPC, thereby decreasing the cholesterol’s condensing effect on the latter. Conversely, the effects of POPS suggest that the area per lipid in pure POPS monolayers is significantly smaller than when present in mixed monolayers at low concentrations. Notably, the observation that the area per lipid in pure POPS monolayers is much smaller than that of the zwitterionic POPC, despite their headgroups being of a similar size and the presence of electrostatic repulsion from the charged PS group, suggests that POPS may be only partially charged in pure monolayers. Alternatively, it may indicate that attractive PS-PS interactions, possibly mediated by bridging countercations, are strong enough to overcome electrostatic repulsion. In mixed systems containing neutral and zwitterionic lipids, where the charge density is low and/or if weaker interactions occur with PC or PE lipids, a larger area per POPS would be observed.

For each system, the $\psi_{d}$ values are determined by the interplay between *A* and the transverse dipole moments $\mu_{⊥}$. Therefore, we now turn our attention to $\mu_{⊥}$, which may be calculated using Equations (1) or (2), as shown in Table 2. For pure systems, a comparison of the values obtained here with those available in the literature [67] was performed in the Section 2. We now address the results obtained for mixed monolayers, comparing them with those expected from the pure systems’ values (including the 373 mD value reported for pure cholesterol by Müller-Landau and Cadenhead [84]), similarly to the previous discussion regarding the area per lipid. The use of Equations (1) and (2) leads qualitatively to the same conclusions. Figure 6, right, shows the values obtained with Equation (1), comparing them with the linear combinations of $\mu_{⊥}$ estimated for pure bilayers, weighted by the mole fractions in the mixture (filled symbols).

It is apparent from Table 2 that the inclusion of cholesterol leads to slight changes in the $\mu_{⊥}$ of POPC and SpM monolayers. For varying cholesterol content, the dipole moment calculated using Equation (1) increases from 546 mD (POPC) to 553 mD (POPC:cholesterol 7:3) and 559 mD (POPC:cholesterol 5:5), possibly indicating the ordering of the POPC dipole induced by cholesterol. The $\mu_{⊥}$ estimated with Equation (1) is essentially unchanged from pure SpM to SpM:cholesterol 6:4. In this case, the effect is negligible, probably due to the fact that the SpM molecules are already highly ordered in the absence of cholesterol. The combination of a small variation in $\mu_{⊥}$ and a large decrease in *A* results in an increase in $\psi_{d\left(30\;\mathrm{mN}/m\right)}^{*}$ for the POPC:cholesterol and SpM:cholesterol monolayers, compared to the systems without cholesterol.

For the POPC:POPE 8:2 mixture, the resulting $\mu_{⊥}$ is lower than that of both POPC and POPE, while, for POPC:POPS 9:1, the opposite behavior (i.e., higher $\mu_{⊥}$ than for pure POPC and POPS) is observed (Figure 6, right). These results reinforce the non-ideality of both lipid mixtures (especially POPC:POPS), and, in agreement with the *A* determinations, they indicate diverging behaviors in the POPC:POPE and POPC:POPS systems.

Regarding the complex mixtures POPC:CHOLesterol:POPE 5:3:2 and POPC:CHOLesterol:POPE:POPS 5:3:2:1, a comparison of the measured $\mu_{⊥}$ values and those expected when assuming linear additivity is carried out with both procedures described above for *A*. Figure 6, right, depicts the values expected for $\mu_{⊥}$ upon replacing 20 mol% POPC with 20 mol% POPE or 30 mol% POPC with 20 mol% POPE and 10% POPS, respectively. Positive deviations are visible in the figure, indicating that the replacement of POPC in POPC:cholesterol 7:3 with POPE and/or POPS, while keeping the cholesterol content fixed, increases the monolayer dipole moment. However, because the corresponding relative increase in *A* is even larger, the dipole potential of POPC:cholesterol:POPE 5:3:2 and POPC:cholesterol:POPE:POPS 4:3:2:1 is actually lower than that of POPC:cholesterol 7:3.

To demonstrate the utility of the membrane potential values obtained in the present work, two applications were selected: the partition coefficients of fluorescent amphiphiles from aqueous media to lipid bilayers and substrate uptake by glucose transporters (GLUT).

**Figure 7:**
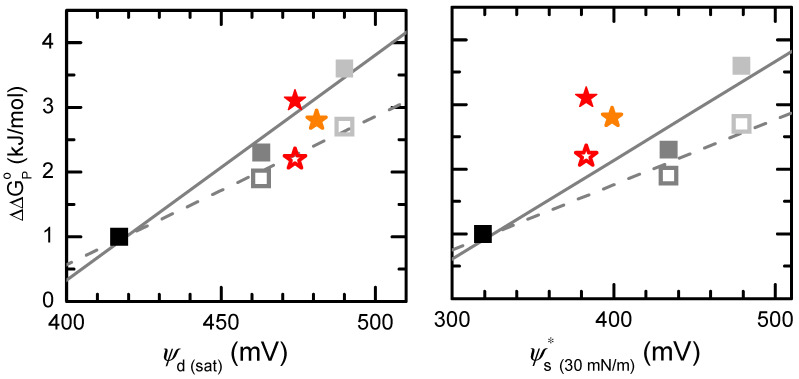
Gibbs free energy difference for partition into different membranes relative to POPC ($\Delta \Delta G_{P}^{{}^{\circ}}$, taken from [13]) as a function of the membrane dipole potential at the saturation pressure (**left**) and at 30 mN/m (**right**). The results for rhodamine-C_14_ are shown as filled symbols and continuous lines, while those for carboxyfluorescein-C_14_ are shown as open symbols and dashed lines. The lines are the best linear fits for the results obtained with the POPC:CHOL membranes (black, gray or white squares for 0, 30 or 50 mol% Chol) and the stars represent the results obtained for the more complex mixtures (POPC:CHOL:POPE 5:3:2 (★) and POPC:CHOL:POPE:POPS 4:3:2:1 (★).

Previously [13], we showed that the orientation of the dipole moment in amphiphilic membrane solutes relative to the membrane dipole potential is relevant for their partition to lipid vesicles. Rhodamine-C_14_ and carboxyfluorescein-C_14_, two amphiphiles that display large chemical similarity but have oppositely oriented dipole moments when inserted in the membrane, display significantly different extents of interactions with the latter. The parallel and anti-parallel alignment of the amphiphile dipole moment and membrane dipole potential leads to increased and decreased (with aggregation in small clusters) solute interactions with the lipid bilayer, respectively. In this study, a linear dependence between the partition free energy from water to a given membrane system relative to that for POPC, $\Delta \Delta G_{P}^{{}^{\circ}}$, and the membrane dipole potential was established. This correlation was limited to systems where values for $\psi_{d}$ were available, namely POPC:cholesterol mixtures. In Figure 7, this is successfully extended, including the newly determined values *Ψ*_d_ for the complex mixtures. These data points follow the linear trends of variation for both amphiphiles when considering $\psi_{d}$ at saturation. Interestingly, poorer correlations were obtained with the values estimated for 30 mN/m lateral pressure. 

For the sake of better control of the dipole moment orientation, the amphiphiles considered in the experiment were suitable fluorescent membrane probes. However, it is anticipated that the same reasoning may apply to interactions of peptides and proteins with membranes, and our previous study already allowed us a possible explanation for the prevalence of protein lipidation at the N-terminal for efficient targeting to the plasma membrane, as well as the tendency of GPI-anchored proteins (usually lipidated at the C-terminal) to form small clusters in the membrane ordered domains. By successfully extending this correlation to a larger set of lipid mixtures representative of actual membrane lipid compositions, the present study reinforces this interpretation.

Several works in the literature describe alterations in GLUT variants upon changing the membrane lipid composition. Suades et al. [87] measured the addition of successive amounts of POPE or POPS lipids to proteoliposomes of rat GLUT5 reconstituted with brain lipids. An approximate twofold increase was observed by adding 18.5% POPS or 35% POPE. In another study [88], POPS, POPE and brain SpM were sequentially added to EggPC to mimic both the inner leaflet and complete plasma membrane mole ratios of these lipids in mammal cells, successively leading to significant increases (both when adding POPS to Egg-PC and POPE to Egg-PC:PS) and a non-significant decrease (when adding SpM to Egg-PC:PS:PE) in GLUT4-mediated glucose uptake. In both studies, the addition of lipids that, according to our measurements, increase or decrease the membrane dipole potential led to augmented or diminished substrate uptake, pointing to its possible relevant effect (certainly along with other factors, such as the membrane fluidity and curvature) on the function of this type of protein.

The dipole potential values determined in this study for different systems may provide insights, albeit tentatively, into other physiologically relevant phenomena. Membrane asymmetry, a fundamental feature of biomembranes, is initially established through asymmetric lipid synthesis and maintained by the slow rate of phospholipid flip-flop and active transport mediated by membrane proteins [33,47,89,90,91,92]. A major consequence of this lipid asymmetry is the generation of transmembrane potentials, which significantly influence the activity of membrane proteins.

While much attention has been given to the potentials arising from charged lipids and charge separation in the aqueous compartments on either side of the membrane, distinct dipole potentials between the two membrane leaflets could generate even larger transmembrane potentials. For example, a transbilayer dipole potential of approximately 100 mV has been experimentally measured in asymmetric membranes prepared from bacterial PE and 1,3-diolein [93] and obtained by molecular dynamics simulations for an asymmetric POPC:POPE membrane [94]. These results are consistent with the difference in the dipole potential obtained in this work for POPC and POPE monolayers (77 mV and 52 mV at $\pi_{\mathrm{sat}}$ and at $\pi_{30\;\mathrm{mN}/m}$, respectively). Encouraged by this agreement, we explored the highly relevant asymmetric lipid composition of the mammalian plasma membrane, modeled by SpM:Chol 6:4 and PC:Chol:PE:PS 4:3:2:1 monolayers for the outer and inner leaflet, respectively [89,95,96]. For this asymmetric membrane, we calculated a transbilayer dipole potential of 117 mV at $\pi_{\mathrm{sat}}$ and 26 mV at $\pi_{30\;\mathrm{mN}/m}$.

The negatively charged POPS in the inner leaflet contributes to the transmembrane potential in two ways: it creates a negative surface potential and increases the dipole potential difference between the two leaflets due to the high dipole moments of monolayers containing low amounts of POPS, as determined in the present work. This transmembrane potential aligns with the direction of the membrane potential observed in cells, which typically ranges from −40 to −80 mV (negative inside). Such potentials are crucial for fundamental processes like muscle contraction and neural transmission [97,98,99]. During apoptosis, the loss of membrane asymmetry—marked by the translocation of PS lipids to the outer leaflet [100,101,102]—increases the dipole potential of the outer leaflet while leaving that of the inner leaflet largely unchanged. This reduces the transmembrane potential and may serve as a signaling mechanism for transmembrane proteins. It also complements the well-established activation of phagocytosis triggered by the exposure of negative charges on the outer leaflets of cells [100,103].

The hypothesis that the distinct dipole potentials of membrane leaflets contribute significantly to these and other fundamental biological processes is compelling and merits further investigation.

## 4. Materials and Methods

### 4.1. Materials

The used 1-palmitoyl-2-oleoyl-*sn*-glycero-3-phosphocholine (POPC), 1-palmitoyl-2-oleoyl-*sn*-glycero-3-phosphoethanolamine (POPE), 1-palmitoyl-2-oleoyl-*sn*-glycero-3-phospho-L-serine sodium salt (POPS), cholesterol (Chol) and egg-sphingomyelin (SpM) were from Avanti Polar Lipids, Inc. (Alabaster, AL, USA). All other reagents and solvents were of analytical grade, or higher purity, from Sigma-Aldrich Química S.A. (Sintra, Portugal). High-quality deionized water, with conductivity of 0.09 μS/cm, was used to perform the experiments.

### 4.2. Monolayer Surface Pressure Experiments

**Figure 8:**
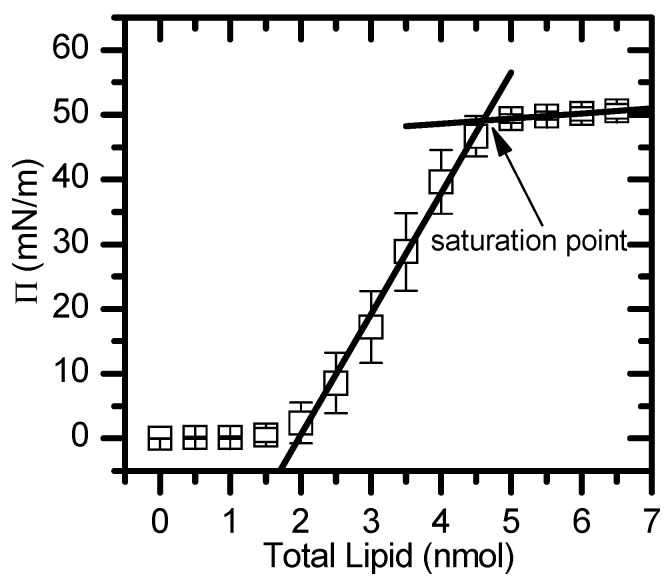
Typical results for a pressure (*π*) vs. total lipid (nmol) isotherm. The two lines represent the plateau at high pressures and the region with steepest slope, respectively. The saturation point corresponds to the interception of both straight lines.

The formation of monolayers at an aqueous surface was monitored through changes in the surface pressure, using Kibron μTrough S equipment, with the addition of a lipid at a constant area and temperature (25 °C). Small aliquots of a chloroform solution containing the lipids of interest (0.5 mM) were carefully spread on the clean surface of an aqueous buffer (NaCl 150 mM, sodium azide 0.02%, EDTA 1 mM and HEPES 10 mM at pH 7.4) and left to equilibrate until the surface pressure reached a constant value. When the addition of further lipids resulted in no visible changes in surface pressure (saturation), the excess lipid formed aggregates in the aqueous subphase and the thermodynamic and interfacial properties were comparable to one leaflet of the lipid bilayer. This procedure allowed for spontaneous lipid stabilization in the aqueous–air interface without applying any lateral pressure [68,104]. The areas per lipid at the saturation pressure (π_sat_) were obtained from the variation in the surface pressure versus the amount of lipid through the interception of two lines, one describing the high-pressure region (small slope) and the other describing the region with the steepest slope (Figure 8).

### 4.3. Measurement of the Potential Difference Across Lipid Monolayers

The potential was measured in lipid monolayers formed at the air–aqueous interface. The system consisted of a high-impedance circuit that connected an ionizing electrode of polonium in the air and a reference (Ag/AgCl) electrode immersed in the aqueous subphase. The monolayers were formed, at the air–aqueous solution interface (NaCl 150 mM, sodium azide 0.02%, EDTA 1 mM and HEPES 10 mM at pH 7.4), by gently adding aliquots of lipids (dissolved in chloroform) with a micro-syringe until the saturation point, after which the further addition of lipids did not induce any change in the measured dipole potential (*V*_lipid_). The difference between the potential of the air–aqueous solution interface without any lipid (*V*_solution_) and the potential arising after the spreading of the lipids (*V*_lipid_) allows the evaluation of the monolayer dipole potential [104]. For the case of zwitterionic lipids, the difference between the measured potentials results from the dipoles of the hydrated lipid monolayer and is directly the dipole potential. For charged lipid molecules, the measured potential reflects the contribution of the water and lipid dipole potentials, as well as from the surface charged potential ($\psi_{0}$) (Equation (3)). Therefore, in order to obtain the dipole potential in monolayers containing charged lipids, we first determined $\psi_{0}$, which was achieved using the Gouy–Chapman theory and the electric charge density (*σ*) at the experimentally determined area per lipid in the saturation pressure (*π*_sat_) (Equation (4)):

(3) $$\psi_{\mathrm{measured}}=\psi_{\mathrm{dipole}}+\psi_{0}$$

(4) $$\sigma^{2}=2000\varepsilon_{0}\varepsilon_{r}RT\underset{i}{\sum }C_{i}\left(e^{\frac{-z_{i}F\psi_{0}}{RT}}-1\right)$$

where $\varepsilon_{0}$ is the vacuum permittivity, $\varepsilon_{r}$ is the relative permittivity, *R* is the ideal gas constant, *T* is the temperature, *F* is the Faraday constant and $C_{i}$ and $z_{i}$ are the concentration and formal charge of the species *i* (considering all charged species in the aqueous phase), respectively.

The statistical analysis of the differences between the dipole potentials was performed using univariate ANOVA, with significance level *α* = 0.05.

## Figures and Tables

**Table 1:** Mean area per lipid and dipole potential obtained for monolayers with different lipid compositions formed at the air–water interface.

	Area per Lipid at *π* = 30 mN/m (Å^2^)	Area per Lipid at *π*_sat_ (Å^2^)	Saturation Pressure (mN/m)	Dipole Potential at *π*_sat_ (mV)
POPC	64.5	49.4 ± 3.4	50	417 ± 12
POPS	55.0 ^a^	44 ^f^	-	465 ± 4.2 ^e^
POPE	56.0 ^b^	42 ^f^	-	494 ± 16
SPM	48.0 ^c^	42 ^c^	-	341 ± 7.1
POPC:POPS (9:1)	69.8	51.0 ± 4.0	51	441 ± 2.5 ^e^
POPC:POPE (8:2)	59.6	45.4 ± 3.0	47	424 ± 15
POPC:CHOL (7:3)	48.0 ^d^	45 ^d^	-	463 ± 6.9
POPC:CHOL (5:5)	44.0 ^d^	43 ^d^	-	490 ± 5.7
POPC:CHOL:POPE (5:3:2)	56.0	46.4 ± 2.7	46	481 ± 1.4
POPC:CHOL:POPE:POPS (4:3:2:1) ^Inner^	61.5	49.7 ± 2.6	49	474 ± 11 ^e^
SPM:CHOL (6:4) ^outer^	39.0 ^c^	-	-	408 ± 6.3

^a^ [64]. ^b^ [65]. ^c^ [29]. ^d^ [66]. ^e^ Final dipole potential obtained from Equation (3). ^f^ Estimated from experimental dipole potential using Equation (1), assuming the same *μ*_⊥_ as for POPC, which was found to be very similar in previous work [24,67]. “inner” and “outer” denote the membrane leaflet mimicked by the corresponding lipid mixtures.

**Table 2:** Dipole potential $\psi_{d\left(30\;\mathrm{mN}/m\right)}^{*}$, calculated for lateral pressure of 30 mN/m using Equations (1) and (2) (the latter considering $\Delta V_{0}$ = 100 mV), and the respective values of the molecular dipole moment component perpendicular to the lipid-water interface, $\mu_{⊥}$. “inner” and “outer” denote the membrane leaflet mimicked by the corresponding lipid mixtures.

	Equation (1)		Equation (2)	
	$\mu_{⊥}$ (mD)	$\psi_{d(30\;\mathrm{mN}/m)}^{*}$ (mV)	$\mu_{⊥}$ (mD)	$\psi_{d(30\;\mathrm{mN}/m)}^{*}$ (mV)
POPC	546	319	415	343
POPS	543	372	426	392
POPE	550	371	439	396
SPM	380	298	268	311
POPC:POPS (9:1)	597	322	461	349
POPC:POPE (8:2)	511	323	390	347
POPC:CHOL (7:3)	553	434	433	440
POPC:CHOL (5:5)	559	479	445	481
POPC:CHOL:POPE (5:3:2)	592	399	469	416
POPC:CHOL:POPE:POPS (4:3:2:1) ^Inner^	625	383	493	402
SPM:CHOL (6:4) ^outer^	379	357	286	370

## Data Availability

The data presented in this study will be sent to interested researchers upon request to the corresponding authors.
